# Sterically Stabilised Polymeric Mesoporous Silica Nanoparticles Improve Doxorubicin Efficiency: Tailored Cancer Therapy

**DOI:** 10.3390/molecules25030742

**Published:** 2020-02-08

**Authors:** Thashini Moodley, Moganavelli Singh

**Affiliations:** Nano-Gene and Drug Delivery Group, Discipline of Biochemistry, School of Life Sciences, University of Kwa-Zulu Natal, Private Bag X54001, Durban 4000, Kwa-Zulu Natal, South Africa; thashinim@gmail.com

**Keywords:** cancer, doxorubicin, drug delivery, mesoporous silica nanoparticles, chitosan, polyethylene glycol

## Abstract

The fruition, commercialisation and clinical application combining nano-engineering, nanomedicine and material science for utilisation in drug delivery is becoming a reality. The successful integration of nanomaterial in nanotherapeutics requires their critical development to ensure physiological and biological compatibility. Mesoporous silica nanoparticles (MSNs) are attractive nanocarriers due to their biodegradable, biocompatible, and relative malleable porous frameworks that can be functionalized for enhanced targeting and delivery in a variety of disease models. The optimal formulation of an MSN with polyethylene glycol (2% and 5%) and chitosan was undertaken, to produce sterically stabilized, hydrophilic MSNs, capable of efficient loading and delivery of the hydrophobic anti-neoplastic drug, doxorubicin (DOX). The pH-sensitive release kinetics of DOX, together with the anticancer, apoptosis and cell-cycle activities of DOX-loaded MSNs in selected cancer cell lines were evaluated. MSNs of 36–60 nm in size, with a pore diameter of 9.8 nm, and a cumulative surface area of 710.36 m^2^/g were produced. The 2% pegylated MSN formulation (PCMSN) had the highest DOX loading capacity (0.98 mg_dox_/mg_msn_), and a sustained release profile over 72 h. Pegylated-drug nanoconjugates were effective at a concentration range between 20–50 μg/mL, inducing apoptosis in cancer cells, and affirming their potential as effective drug delivery vehicles.

## 1. Introduction

Nanotherapeutics postulates the use of nanotechnology for the alleviation of a variety of diseases, by specifically diversifying treatment options and reducing conventional treatment- associated side effects [1]. This has inspired the design of various nanocarriers that aim to reduce pure drug concentrations and dosing frequencies, commonly associated with the onset of toxicities and drug resistance, by providing a therapeutically efficient, biocompatible administration route [2,3]. The appeal of nanoparticles (NPs) extends to their size, relative biosafety and multi-functionality that can be adapted for disease-specific models [4,5]. They are optimally designed to cross physiological barriers with ease, are classed as generally immunological compliant, and can access a variety of tissues [6,7]. They also allow for the reformulation and stabilisation of toxic drugs, diagnostic elements, and corrective genes, making them clinically and commercially beneficial [4].

Current research into material engineering and nano-architectural design have seen the development of an array of NPs, with MSNs emerging as a fore-runner in biomedical research. MSNs have a highly flexible and tunable framework, that is biodegradable and biocompatible in biological systems [8,9], together with a narrowly distributed 2D hexagonal porous network [10]. MSNs possess a large active surface area which can be selectively polymerised or functionalised for stimuli-responsive purposes [11], tunable pore size and large pore volumes for the loading and controlled release of the cargo, and have shown favourable tolerance levels both in vitro and in vivo [12,13,14].

MSNs are being extensively researched as theranostic devices for diseases, especially for cancer therapy [15]. Conventional cancer treatment options such as surgery, radiotherapy and chemotherapy [16], have not been fully effective, resulting in snowballing recurrence rates and depression in the quality of life [17,18]. Unpleasant side-effects are often linked to the anti-neoplastic drugs used, which act by inhibiting cellular mechanisms of DNA replication that are up-regulated in cancer cells. These cytostatic or cytotoxic compounds usually have low bioavailability and are thus administered at high dosages or for prolonged dosing intervals, leading to systemic side effects at non-specific sites [19,20]. 

Doxorubicin (DOX) remains one of the most efficient anthracycline drugs available and is used in the treatment of diverse cancers, including breast, cervical, bone, gastric and leukaemia [21,22,23]. Despite its popularity, its low solubility [24,25,26], coupled with increased dosing frequencies [27,28,29] has resulted in many associated side effects, including cardiotoxicity [30,31,32], myelosuppression [33,34], induced vomiting with nausea [35,36], and alopecia [18,37]. Critical evaluation of these detrimental side-effects that become more pronounced as dosing durations increase has concluded that both chronic and acute DOX-induced cytotoxicity can be largely reduced with improved and targeted administration routes [21,38]. This has led to the production of the commercially available liposomal DOX formulation, Doxil^®^, which enhanced the drugs’ performance and reduced some of the side-effects [39].

In this study, an optimised MSN with a large active surface area and large pore volume, was selectively functionalised with the organic polymer chitosan (C) and inorganic polymer polyethylene glycol (P), to create a hydrophilic, polyelectrolyte complexed superficial layer, that allowed for the transport of the hydrophobic drug, DOX. MSNs functionalised with chitosan and PEG, as in this study have been previously reported to successfully deliver the anticancer drug 5-fluorouracil to mammalian cells in culture. The authors reported favourable drug loading, drug release and increased anticancer activity (>50%) in Caco-2, MCF-7, and HeLa cells in vitro when compared to the non-cancer, HEK293 cell line [40]. Similarly, these non-toxic and biocompatible drug delivery vehicles may also improve the therapeutic efficiency of DOX in vitro.

## 2. Results

### 2.1. Size and Morphology

**Figure 1:**
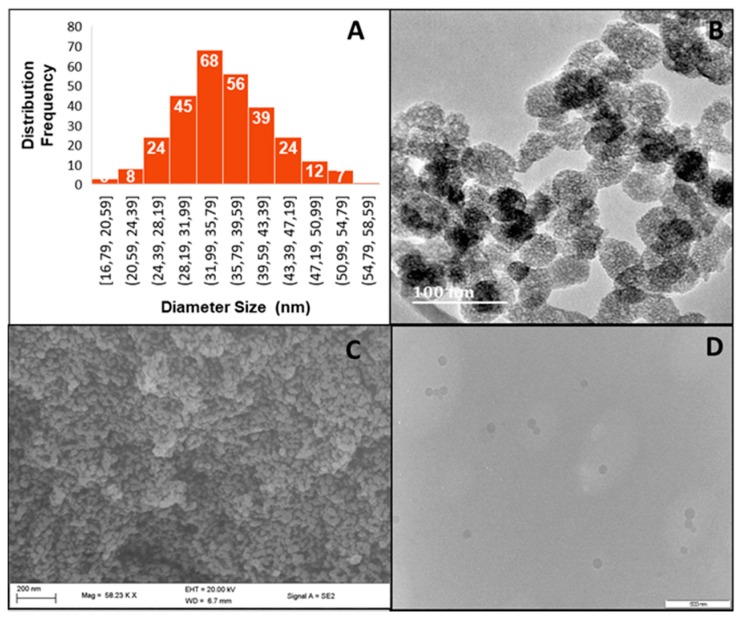
(**A**) Size distributions of MSNs from TEM images, (**B**) HRTEM image of MSN (Bar = 100 nm), (**C**) SEM image of 2% PCMSN (bar = 200 nm), and (**D**) DOX loaded 2% PCMSN (50 kV) (Bar = 500 nm.).

With the potential hemocompatibility [41], biocompatibility [42], and ultimate pharmacokinetic fate [43] of MSNs in mind, the Ströber method with modifications was used [44]. Electron microscopy (Figure 1) revealed MSNs from 36.09–40.75 nm in size, which increased upon DOX loading to a maximum of 59.98 nm. Figure 1 further shows spherical monodisperse MSNs, the polydispersity index (PDI) [45] of which were calculated from TEM and NTA using the following equation:

(1) $$PDI=\;{\left(\;\frac{\sigma }{D}\;\right)}^{2}$$

where σ is the standard deviation, and D is the mean diameter obtained from TEM or NTA. The PDI’s from both calculations were generally below 0.05, indicating a relatively stable monodisperse population of MSNs, except for the 5% PCMSN-DOX which was polydisperse.

Post-grafting was utilised to functionalise the surface of the MSNs, by protonating their outer surface and increasing their hydrophilicity [46]. This contributed to the enlarged hydrodynamic sizes from NTA (Table 1). Furthermore, polymer coating conferred a large hydrophilic charge surrounding the functionalised MSNs, with polyethyleneglycol covering some of the positive charges provided by the amine groups in chitosan. Hence, the zeta potential of the PCMSNs was less positive than that of the CMSNs (Table 1). 

DOX is typically uncharged and hydrophobic with an ionizable primary amine (pKa of 8.3) [47]. Once internalised in the hydrophobic core of MSNs, the particle swells in size, as seen under TEM and NTA (Table 1). The zeta potential remained positive but closer to neutral in aqueous solutions. This may allude to DOX selectively binding to the outer surface of the MSNs, as well as loading internally. Furthermore, the swollen MSN may have also released DOX in the aqueous medium, hindering its Brownian movement under NTA, resulting in a lower zeta potential.

**Figure 2:**
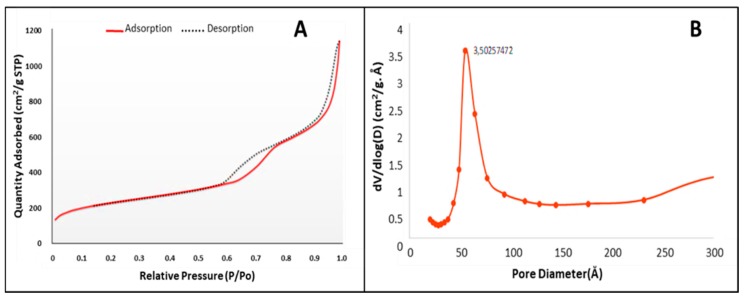
(**A**) Nitrogen adsorption-desorption isotherm and (**B**) pore size distribution of MSN.

Nitrogen adsorption-desorption studies provided data defining the surface area, pore morphology and pore volume of the MSNs. The adsorption-desorption isotherm (Figure 2), is a type IV isotherm and displays two defined hysteresis loops at P/P_0_ = 0.6 − 0.75 and P/P_0_ = 0.87 − 0.9, indicating a mesoporous silica material with narrowly distributed pores spaced at 3.5 nm, and well-defined capillary condensation and desorption. The hysteresis loops had a characteristic H1 shape, with a sharp slope indicating a cylindrical pore shape and a pore volume of 1.74 cm^2^/g. Swelling of non-rigid pores may account for the hysteresis loop shape at low pressure. The pore size diameter, calculated according to Barrett, Joyner, and Halenda (BJH) was 9.8 nm (98 Å) (from desorption branch), with a cumulative surface area of 710.36 m^2^/g. 

### 2.2. MSN Surface Modification

MSN surface modifications were accomplished by post-grafting of combined polymers to the outer surface of the MSN. The polymerisation of MSN is a first-line defence against diversified environmental conditions and in vivo immunological responses. The incomplete capping, which selectively covers the pore entrance allows for controlled drug release at the target site and endosomal escape. The silanol groups that cover the MSN surface and impart a negative zeta potential at pH ~7 were first functionalised with chitosan, followed by the incorporation of a 2 % or 5% polyethylene glycol (PEG) that were linked to the amines of the chitosan. The hydrophilic layer around the MSN is important for enhanced uptake of DOX.

**Figure 3:**
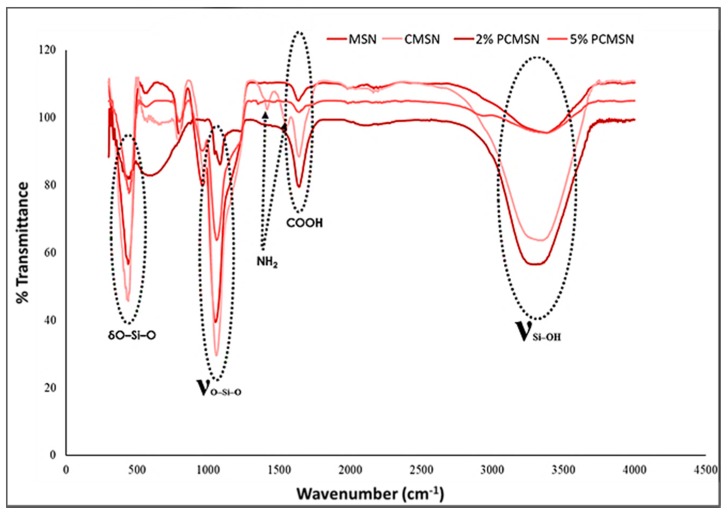
FTIR spectra of MSN, chitosan-MSN (CMSN), 2% PEG-chitosan-MSN (2% PCMSN), and 5% PEG-chitosan-MSN (5% PCMSN).

FTIR analysis provided a spectral confirmation of distinguishing vibrational peaks signifying the addition of the polymers onto the surface of the MSN (Figure 3). A peak at 1645 cm^−1^ related to the C=O vibration of COOH, and two N-H peaks at 1413 cm^−1^ and 1547 cm^−1^ indicated the binding of PEG and chitosan, respectively, onto the MSN surface. All samples had strong bands at 430 (O–Si–O), 1058 (Si–O–Si), and 3300–3500 cm^−1^ (Si–OH), confirming the presence of the SiO_2_ inorganic phase. Peaks were assigned as reported in the literature [48,49].

These results were confirmed by the elemental analysis provided by Energy Dispersive X-Ray Spectroscopy (EDX)/SEM (Table 2). MSNs elemental composition was distinctly constituted of silicon dioxide, and due to the addition of chitosan and PEG, the presence of carbon with oxygen and silica was apparent. This simplistic mineralogical break down of the constituents of the MSNs confirmed the FTIR results indicating that favourable surface modification was achieved.

### 2.3. Doxorubicin Loading

**Figure 4:**
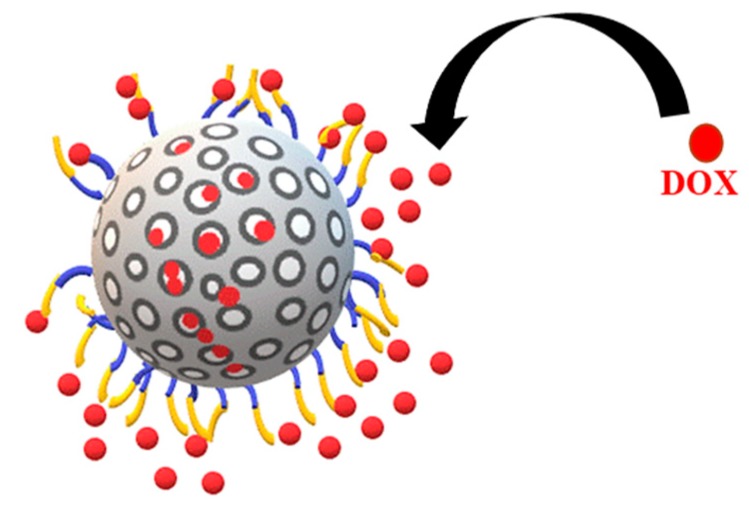
Representation of polyethylene glycol and chitosan-coated MSN loaded with DOX.

The favourable surface modification led to a significant uptake of DOX into both PCMSNs of 93.32% and 97.85% respectively (Table 3). The MSNs were well-dispersed and once dried appeared as a red powder. The loading of DOX was considerably higher than that previously reported by the authors for 5-fluorouracil (15–18%), which was attributed to this formulation being highly polydisperse with 5-fluorouracil loading being controlled by the stability of the MSNs [40]. Figure 4 is a schematic representation of DOX loading onto the MSN.

### 2.4. Doxorubicin Release and Pharmacokinetic Modelling

**Figure 5:**
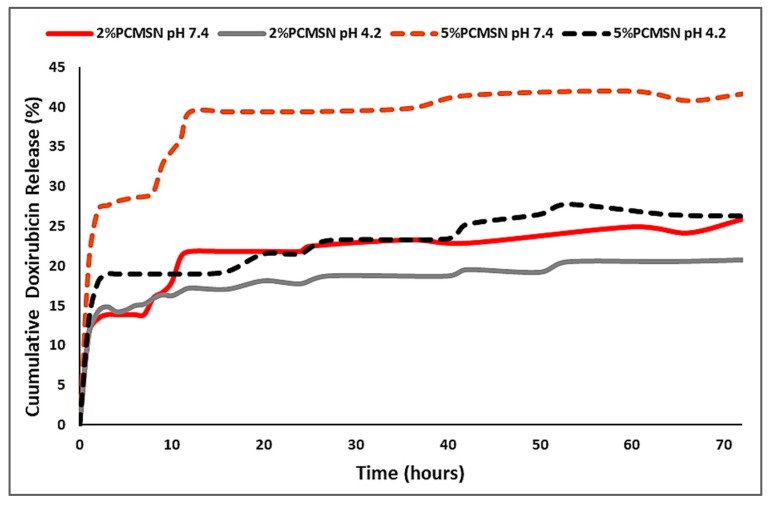
Drug release profiles of DOX at pH 7.4 (red/dark red series) and pH 4.2 (grey/black series) for 2% PCMSNs (solid line) and 5% PCMSNs (dashed line).

In vitro release studies for the 2% PCMSN and 5% PCMSN DOX-loaded formulations at physiological pH 7.4 (blood plasma serum and extracellular fluid) and pH 4.2 (endosomal/lysosomal intracellular trafficking vesicles, and acidic tumour microenvironment) are illustrated in Figure 5. At pH 7.4, both polymeric delivery vehicles exhibited a gradual steep release from 0 to 12 h. This may be attributed to the hydrophobic DOX molecules adsorbed on the outer surface of the MSN formulation interacting with water molecules and following a concentration-gradient diffusive pattern. After 12 h and up to 50 h, a sustained gradual release pattern was observed. The 5% PCMSN formulation displayed a higher percentage release, which may be attributed to a higher number of adsorbed DOX onto the more hydrophilic and PEGylated layer surrounding the MSN. Under acidic conditions, the release was sustained in both formulations, with 20% or more of the drug being released from both NPs. This sustained drug release profile was similar to that achieved previously for 5-fluorouracil [40]. However, there was a greater release of DOX (20%) in this study compared to that of 5-fluorouracil (>10%) at acidic pH, but a lower release of DOX (>40%) over 5-fluorouracil (>70%) at physiological pH.

The conventional drug release kinetic models tested were zero order, first order [50], Higuchi [51], Hixson- Crowell [52], and Korsmeyer- Peppas [53]. The contribution of diffusion and erosion to the release patterns seen was evaluated and quantified using the Kopcha model [54]. In this model, the constants A, representative of diffusion and B, representative of erosion, were used to illustrate mathematically which of these two factors affected release more. According to literature, when A/B = 1, diffusion and erosion is equal. However, when A/B < 1, erosion dominates over diffusion, and conversely for A/B >1, the diffusion is not affected by erosion.

The best release model was selected based on the correlation coefficient (R^2^) obtained and release exponents that described the release patterns observed are defined based on the equations below:

Zero Order model [55]:

(2) $$M_{t}=\;M_{0}+\;k_{0}t\;$$

First Order model [56]:

(3) $$logM_{t}=\;\log M_{0}+\;\frac{k_{1}t\;}{2.303}$$

Higuchi model [51]: This model assumes release from an insoluble matrix as a time-dependent progression in which Fickian diffusion is supposed:

(4) $$M_{t}=\;k_{H}\sqrt{t}$$

Hixson- Crowell model [52]: This cube root model describes release by dissolution and accounts for changes in the surface area and diameter of the particle:

(5) $${\left(M_{t}-\;M_{\infty }\right)}^{1/3}=\;k_{HC}.\;t$$

Korsmeyer-Peppas model [53,57]: Follows release from a spherical polymeric system in which there may be diffusion or erosion:

(6) $$\frac{M_{t}}{M_{\infty }}=\;k_{KP}\;.\;t^{n}$$

Kopcha model [54]: is used to define the amount of diffusion and erosion, and its effects on the release rate:

(7) $$M_{t}=\;A\;.\sqrt{t}+Bt$$

where M_0_, M_t_ and $M_{\infty }$ represent the amount of drug dissolved at time zero, time *t*, and at infinite time, respectively. The kinetic constants are represented by k and subscripted with their model initial. 

The release exponent *n* is derived from the Korsmeyer-Peppas model and was used to define the release mechanism. When *n* = 1, the release is zero order; when *n* = 0.43, the release is best described as Fickian diffusion where there is no relevant deformation or stresses during drug release. When 0.43 < *n* < 0.85, the release is through anomalous diffusion where there may be swelling or stress during drug release, and these structural changes may be due to temperature, activity or structural dimension related fluctuations. If *n* > 0.85 there is Case II transport [58,59]. Models which have a correlation coefficient higher than 75 % are generally considered as suitable models [50,60]. The above models used are well established models used to evaluate the release of drugs from nanocarriers mathematically. The correlation coefficient is typically used to explain which model best fits into the drug release data.

In this study (Supplementary Tables S1–S3, Supplementary Figure S1), the 2% PCMSNs fitted the criteria for the first order model, but the most appropriate model fit was with Higuchi’s diffusion release model and Kopcha’s model, indicating that release occurred mostly through diffusion, with minimal erosion effects. Higuchi’s model states that the time taken to release 50% of the total amount of the drug within the matrix corresponds to 10% of the time taken to dissolve the final traces of drug (cube root of time model) [51,61]. This was in accordance with Figure 5, where an initial “burst release within the first 12 h was followed by a slow release for the next 60 h, under both pH conditions. The 5% PCMSN, fitted the Higuchi’s model as well as the Korsmeyer-Peppas and Kopcha’s models (Supplementary Table S3), where drug release mechanisms were shown to occur by Fickian diffusion, with minimal erosion or swelling of the matrix [45]. According to Korsmeyer-Peppas, all DOX -loaded MSNs displayed a quasi Fickian diffusion release pattern (*n* < 0.43). The Kopcha model fitted all four models with high correlation values, with all A values recorded being much higher than the calculated A/B values. This indicated that diffusion was predominant over erosion for the duration of the 72-h drug release period [53,58]. 

### 2.5. Cell-Based Cytotoxicity Studies

**Figure 6:**
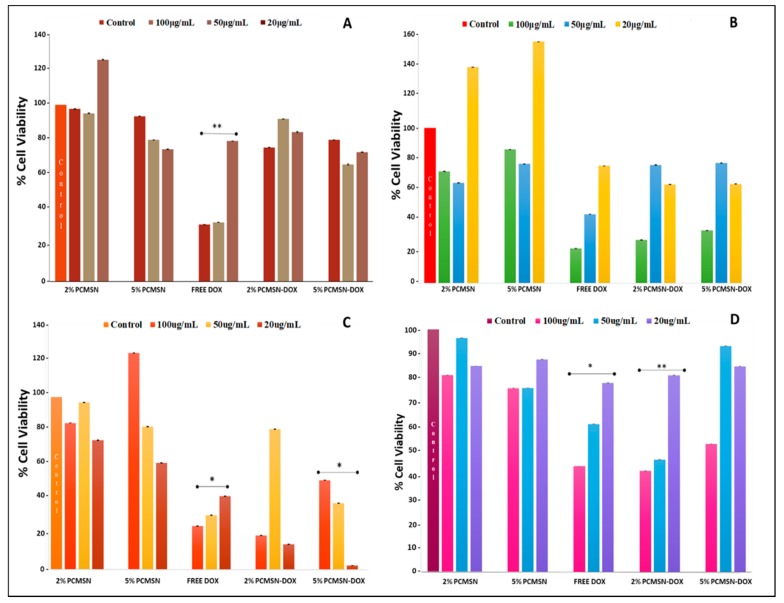
MTT cell viability assay of MSNs and DOX-loaded MSNs administered at various concentrations (20, 50 and 100 μg/mL) in (**A**) HEK293, **(B)** Caco-2, (**C**) MCF-7 and (**D**) HeLa cells for 48 h. Data is represented as means ± SD (*n* = 3). * *p <* 0.05, ** *p <* 0.01 were considered statistically significant.

The use of specific mammalian cell-models is a well-established technique that is utilised to screen the biocompatibility of nanoparticle formulations and analyse their bioavailability and pharmacological response to the delivery of an anticancer drug. Cytotoxicity is a measure of the influence of an administered drug at varying concentrations, and its effect on cellular physiology. From Figure 6 A, it is evident that none of the MSN formulations elicited a significant cytotoxic response in the HEK293 cells Free DOX did cause a reduction in cell population after 48 h, indicating the effectiveness of this anti-neoplastic drug and its potential toxicity. 

For the Caco-2 cells (Figure 6B), the higher concentration of administered MSN (100 μg/mL) produced a reduction of cell number by 50%, indicating that this dosage was therapeutically significant. In the MCF-7 cells (Figure 6C), a more pronounced effect was seen, especially with the lower dosage. In the Hela cells (Figure 6D), a higher dosage of drug-loaded MSNs (50–100 μg/mL) was needed to see a treatment response in the cell population. However, free DOX in comparison to MSN-DOX failed to effectively reduce HeLa cell numbers at the higher concentrations, hinting at the inheritance of possible drug transport pumps or resistance mechanisms that may reduce the effectiveness of conventional chemotherapy drugs. The IC_50_ values calculated (Table 4) from the cytotoxicity assay were used further in the apoptotic studies.

#### Apoptosis and Cell Cycle Analysis

**Figure 7:**
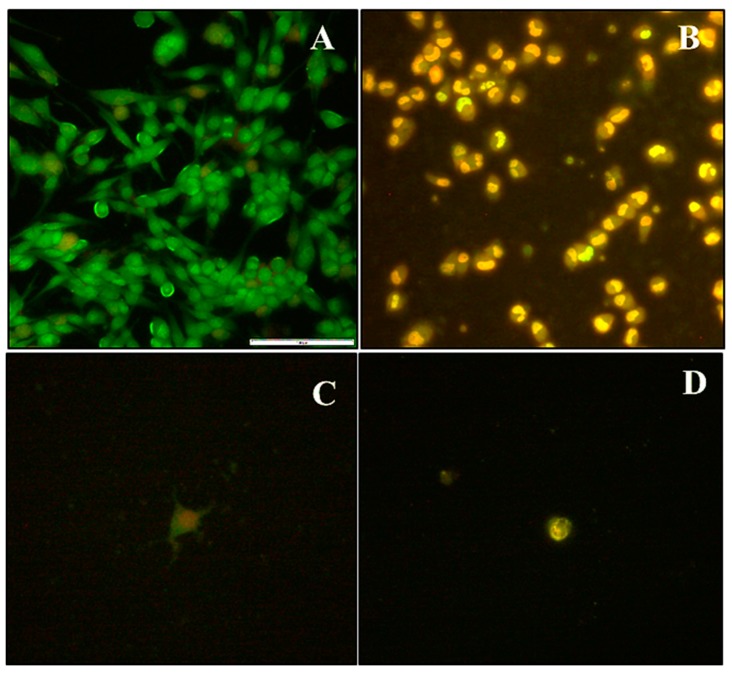
Selected fluorescent micrographs of dual acridine orange/ethidium bromide-stained cells showing induced morphological changes in (**A**) HEK293 cells, (**B**) Caco-2 cells treated with DOX loaded 2% PCMSN (**C**) MCF-7 cells treated with DOX loaded 5% PCMSN, and (**D**) HeLa cells treated with DOX loaded 5% PCMSN at 20× magnification.

The apoptotic images obtained (Figure 7), were used to reinforce the cytotoxicity data, and correlate the influence of DOX-MSN treatment with programmed-cell death mechanisms induced in the cell lines tested. 

**Figure 8:**
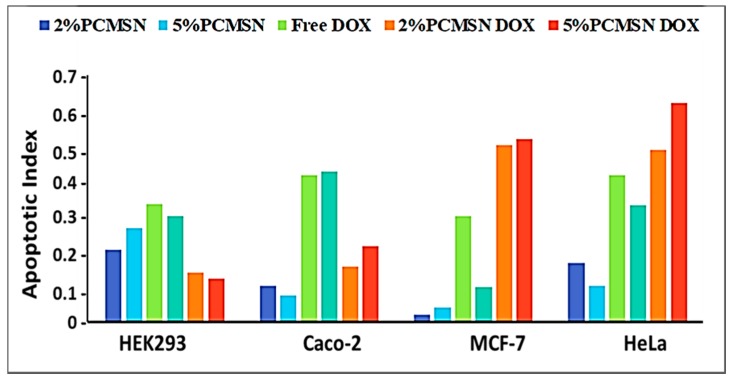
Apoptotic indices calculated from fluorescent micrographs taken of each cell line treated with MSNs, drug and DOX-loaded MSNs.

HEK293 cells treated with MSN drug formulations had low apoptotic indices (Figure 8), indicating treatment exposure did not effect a significant change in cellular functioning. In Caco-2 cells, the free drug was potent and elicited a significant response with most cells undergoing apoptosis. In the HeLa and MCF-7 cells, mostly apoptotic cells were visible when treated with both 2% and 5% PCMSN-DOX, with many cells rounding off and floating off the wells (Figure 7C,D). Hence, the cell population is sparse in the captured images. This change in the cell morphology may be linked to altered differentiation and a change in cell cycle behaviour, which was investigated further by cell cycle analysis.

**Figure 9:**
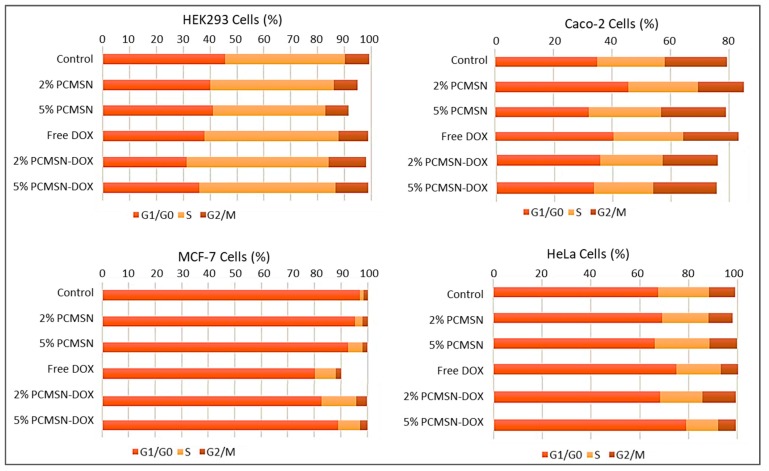
Cell cycle distribution in HEK293, Caco-2, MCF-7, and HeLa cells.

Cell cycle analysis (Figure 9), showed that in the MCF-7 cells, there was an increased distribution of cells in the G_0_/G_1_ and S phase after treatment with DOX-loaded MSNs. In the HeLa cells, treatment with 2% PCMSN-DOX showed a slight decrease in the number of cells in the S phase and a slight increase in cells numbers in the G_2_/M phase. Upon treatment with 5% PCMSN-DOX, there was an increase in the distribution of HeLa cells in the G_0_/G_1_ phase. In the Caco-2 cells, there was no notable shift in cell distribution, however, treatment with DOX-loaded MSN produced a reduction in the cell population (%), indicating that cells had fragmented after undergoing apoptosis (Figure 7B).

## 3. Discussion

The localised tumour microenvironment is characterised by extreme metabolic processing and rapid, uncontrolled replication and therefore possesses singular features that can be exploited for site-specific targeting [62,63]. A typical tumour undergoes rapid and progressive angiogenesis to form abnormal vasculature for the increased supply of nutrients and oxygen [64]. These blood vessels consist of flattened endothelial cells, with large gaps between the basement membranes, allowing for molecules larger than 40 kDa to accumulate mostly in the tumour tissue [65], without immune system interference [62,66]. Along with this passive targeting, polymerisation of the nanoparticle has been found to regulate cellular uptake rates, prolong in vivo circulation times and prevent rapid renal clearance and MPS (mononuclear phagocyte system) escape [67,68,69,70].

There are noted advantages of polyethylene glycol (PEG) grafting onto the surface of NPs including silica nanomaterials, with studies suggesting that polyethylene glycol coating aids in the escape of phagocytosis by binding specific serum proteins such as dyopsonins, leading to higher hemocompatibility with red blood cells [41,71,72,73], and increasing the circulating half-life of PEG-MSNs. PEG-MSNs of smaller size were effective in evading immune responses in mice models and were degraded slowly with no systemic or tissue-specific toxicity noted for up to a month after treatment [43]. Factors such as size, morphology and favourable surface modifications contribute to cellular uptake, biocompatibility, prolonged circulation time and pharmacokinetic fate [74]. 

The size of a PEG-coated NPs should optimally be between 20-200 nm in diameter in order to minimise opsonisation [75,76]. Positively charged MSNs were found to have enhanced endocytosis by electrostatically interacting with the negatively charged cell membrane [6,77], while negatively charged NPs can escape the endosome efficiently, probably by the “proton sponge effect” [74]. The physio-chemical properties of the MSNs in this study and their drug-loaded formulations were prone to agglomeration, which could have affected their zeta potential. Small monodisperse MSNs were produced, which increased in size after functionalization. Post-grafting is a cheap and simplistic surface modification method that uses fewer reagents, and results in reproducible batch-to-batch results, as seen with a monodisperse MSN distribution being maintained post-modification. Electron microscopy images of MSN drug nanoconjugates taken up to a year after preparation and storage, showed no significant matrix degradation or pore collapse, further indicating the stability of these MSNs. 

The large active surface area (710.3616 m^2^/g) of the MSNs once polymerised attained a weakly positive charge and an increase in hydrophilicity as indicated by a 2-fold increase in the hydrodynamic size. This hydrophilicity favourably influenced the loading of the hydrophobic chemotropic drug DOX into the mesoporous framework. The large cylindrical pore size (~9–10 nm) and pore volume (1.743321 cm³/g) allowed for the easy uptake of DOX into the silica framework, resulting in a high loading capacity of 93% and 98 % in the 2% PCMSN and 5% PCMSN formulations, respectively. This was much higher than a previous report on DOX loading into nanoparticles using a similar method [78]. Due to the many DOX molecules adhered to the PEG coating on the interfacial surface of the MSN, a burst release under physiological conditions (pH 7.4), was evident during the drug release studies.

Kinetic modelling of drug release can be used to predict the possible release mechanisms involved, thereby allowing for potential improvement to the formulation before further application [60]. To date, no generalised and definite kinetic behaviour model of drug release from NPs have been reported in the literature [79]. All model behaviours applied utilised the linearization of release, which may alter characteristic deviations due to the material type, matrix specifications, fluctuating bath conditions, concentration gradients and interaction between the material, drug and solvent. Thus, the use of non-linear regression models and alternate models may be utilised for future use, but these may still be restricted to in vitro conditions [55]. The Higuchi models describe drug release from a matrix system but required that the system contain a high amount of the drug, diffusion to occur in one dimension, drug particles to be smaller than the matrix and that there should be no swelling and dissolution. The diffusion is constant with the sink conditions being maintained [51,61]. 

Drug release profiles typically fitted well into Higuchi’s, Korsmeyer-Peppas [53] and Kopcha’s [54] models. The release of DOX from the functionalized MSNs can be described as undergoing rapid Fickian diffusion with no friction effects within the first 12 h, during which most of the drug was released into the solution. This was then followed by a slower, controlled release of the drug. Thus, as the weight of the MSN was reduced, and the medium became saturated with the drug, the release profile reached a plateau. However, according to the kinetic modelling, the matrix was not eroded as diffusion was still favoured, indicating that the concentration of the drug in the bath was saturated where free movement was inhibited; or a hydration cloud surrounded the hydrophilic MSN as water molecules replaced DOX molecules within the matrix, preventing the rapid movement and the final elimination of DOX, as seen in the initial hours of release [55,80]. 

Kopcha’s model [54] indicated that the MSN matrix degradation was mostly through diffusion, with minimal erosion and degradation of the drug release matrix at the varying pHs. This is an integral parameter for a drug delivery vehicle, as the delivery of the cargo should be relatively independent of structural degradation. Optimal drug formulation is crucial for further commercial and clinical applications. These formulations must induce matrix stability and promote a sustained release of the drug over time, with minimal erosion [81,82]. Although kinetic modelling revealed promising advantages and attributes, further investigation of MSN’s potential in vivo, and its influence on the structure and release patterns need to be evaluated [83]. 

Silica is “generally recognised as safe”, with abundant usage in consumables and cosmetics reported. The assessment of the cytotoxicity of DOX-loaded MSNs in established disease-specific mammalian cell models is necessary. MSNs possessed a weakly positive, almost neutral charge once loaded with DOX, with the average release of the drug by diffusion contributing to the distortion of the morphology and the size of MSN. This favourably affected the biocompatibility and uptake of the MSNs into the cells. There were no cytotoxic responses induced with treatments of DOX loaded MSNs in normal HEK293 cells, suggesting potential selective targeting to cancer cells. The DOX-MSN formulations induced extreme apoptotic events in the Caco-2, MCF-7 and HeLa cells, with most of the cells losing their extensions, undergoing nuclear condensation, membrane blebbing, and thus displaying apoptotic signals [84,85,86]. The apoptosis assay corroborated the results of the cytotoxicity assay, as also evidenced in other studies [87]. These results were further confirmed by cell cycle analysis, with a large percentage of DOX-treated MCF-7 and HeLa cell events defined as cell debris, indicating fragmented cells that had undergone apoptosis or necrosis. The study of the cell cycle, including its transition and arrest, is an important parameter used to deduce whether DNA damage has occurred, and whether normal cell repair mechanisms were activated or whether apoptosis had been induced. The number of cells distributed within the G1/S or G2/M phases is generally suggestive of cells that have initiated the DNA damage response (DDR), and appropriate repair pathways in response to the addition of DNA-targeting chemotropic drugs.

Cell cycle progression is monitored and tightly regulated by cyclins, cyclin-dependent kinases (CDKs), and critical regulatory proteins, including the tumour suppressor, p53 [88,89]. With induced DNA damage from DOX and intercalation and inhibition of thymidylate synthase, cellular checkpoints are activated to arrest cell progression to the replication phase [90,91]. However, cell death during mitosis is activated by caspase-2 and prevents defective or damaged DNA from being replicated and passed on to the daughter cells [92,93,94,95]. Alternatively, checkpoint 1 (Chk1) activation occurs mostly during the S or G2 phases, and recruits repair mechanism pathways, and prevents progression to the M phase [91]. For MCF-7 cells, an increase in the S phase indicated that a percentage of the cells had been marked for apoptosis after treatment with DOX-loaded MSNs. This was confirmed with the recorded morphological changes associated with apoptosis in most of the cell populations. For the HeLa cells, free DOX showed no significant change in the cell distribution between the cell cycles, suggesting the poor performance of DOX in vitro. However, when DOX was dissolved in the hydrophilic matrix of the MSN, there was an increase in cytotoxic and apoptotic events, with the cell cycle confirming a high percentage of debris and increase in cells within the G2 phase checkpoint. 

For the Caco-2 cells, only high concentrations of the DOX-MSNs elicited a cytotoxic effect. The cytotoxicity profile further suggested that passive internalisation routes were utilised and that a controlled drug release mechanism within the cellular environment as shown by the drug release studies and kinetic modelling occurred [96]. Overall, the MSN-DOX nanoconjugates did not induce significant cytotoxicity or apoptotic events in the HEK293 cell population. Furthermore, the bioavailability of DOX was increased when formulated with MSN, inducing cytotoxicity and apoptosis more effectively, suggesting a synergistic effect of the MSN conjugated to the drug.

## 4. Materials and Methods 

### 4.1. Materials

Tetraethyl orthosilicate (TEOS, Si(OCH2CH3)4), Triton X-100 (TX100), cetyltrimethyl-ammonium bromide (CTAB, 99%), polyethyleneglycol_2000_ (PEG_2000_), chitosan (75–85 % deacetylated), sodium tripolyphosphate (TPP), Tween 20, ammonia solution (28–30%), sulphuric acid, sodium carbonate (Na_2_CO_3_), doxorubicin hydrochloride (DOX, Mw: 579.98 g mol^−1^), and deuterium oxide, were all purchased from Sigma Aldrich (St Louis, MO, USA). Eagle’s minimum essential medium (EMEM), fetal bovine serum (FBS), penicillin/streptomycin solution (10,000 U/mL), and trypsin−EDTA (0.25% trypsin, 0.1% EDTA) were obtained from Lonza (Verviers, Belgium). Phosphate-buffered saline (PBS) tablets were purchased from Calbiochem (Darmstadt, Germany) The MTT salt (3-(4,5-dimethylthiazol-2-yl)-2,5-diphenyltetrazolium bromide) and trichloroacetic acid (TCA) were purchased from Merck (Darmstadt, Germany). All sterile plasticware for tissue culture were obtained from Corning Inc. (Corning, NY, USA. All other reagents were of analytical grade with 18 MΩ water being used throughout (Millipore, Molsheim, France).

### 4.2. Synthesis of Mesoporous Silica Nanoparticles (MSNs)

MSNs synthesis using a sol-gel reaction was adapted from literature [44,97], and as described previously by the authors [40]. Approximately, 500 µL tetraethyl orthosilicate (TEOS) was added to a solution containing cetyltrimethylammonium bromide (CTAB, 100 mg in 48 mL ddH2O) and 350 µL of 2 M NaOH, and incubated for 2 h. The MSN product was obtained by centrifugation (4000 rpm, 30 min), washed with absolute ethanol and subsequently with 18 MΩ water. Residual CTAB was removed by acidic methanol (20 mL methanol, 1 mL 37 % HCl) reflux at 80 °C overnight, followed by centrifugation. The pelleted MSNs were calcined at 70 °C over 24 h to remove unreacted material. 

### 4.3. MSN modification with Chitosan and Polyethyleneglycol

MSN modification was adapted from literature [98,99] and was described previously by the authors [40]. Approximately, 200 mg of MSNs and 15 mg of chitosan (C) in 40 mL of acetic acid (10 % *v*/*v*), and stirred at ambient temperature over 24 h. The CMSNs were then centrifuged, washed with absolute ethanol and thereafter with deionized water, and dried at 60 °C for 24 h. For modification with polyethylene glycol (P) and chitosan (C), 22.5 mg of C and 179 mg or 449 mg of polyethylene glycol 2000 were added separately to dilute acetic acid (30 mL, 2%; pH 4.6), followed by the addition of 7.725 mg of TPP (15 mL in 18 MΩ water). The mixture, was then added to 300 mg of MSN, stirred at room temperature for 24 h, and the final products (2% and 5% PCMSN) were collected by centrifugation (1000 rpm, 30 min), washed and dried at 60 °C for 24 h.

### 4.4. Doxorubicin (DOX) Loading

Approximately, 100 mg of the respective PCMSNs and CMSN were added to saturated solutions (5 mg/mL, 5 mL) of DOX with stirring for 30 h, to allow the drug to enter the MSN mesoporous framework [40,100]. Aliquots (100 μL), of the drug solution, was removed at 0 and 30 h, centrifuged, and the supernatant analysed using a UV spectrophotometer (Nanodrop oneC, Thermo-Fischer Scientific Inc., Waltham, MA, USA) at 488 nm. The precipitate was returned to the solution after analysis. The DOX-loaded MSNs were obtained by centrifugation, washed and dried at 50 °C for 24 h. The drug loading capacity was calculated using the following equation [82]:

(8) $$\mathrm{Loading}\;\mathrm{capacity}\;\left(\mathrm{wt}\%\right)=\frac{\mathrm{Mass}\;\mathrm{of}\;\mathrm{drugs}\;\mathrm{in}\;\mathrm{MSN}}{\mathrm{Initial}\;\mathrm{Mass}\;\mathrm{of}\;\mathrm{MSN}}$$

### 4.5. Electron Microscopy and Energy Dispersive X-Ray Spectroscopy

The ultrastructural morphology of the MSNs and their drug nanoconjugates were determined as previously [40], using transmission electron microscopy (TEM; JEM 1010, JEOL, Tokyo, Japan), and high-resolution transmission electron microscopy (HRTEM; JEOL JEM 2100 with ECSI 10 digital micrograph software) at an accelerating voltage of 100 kV. TEM and HRTEM samples (5 mg MSN in 5 mL ethanol) were placed onto carbon-coated grids and air-dried. Images were captured, and nanoparticles were individually measured and expressed in mean size distribution graphs. Scanning Electron Microscopy (LEO 1450 SEM, Carl Zeiss, Oberkochen, Germany,with SmartSEM software Version 5.03.06), was employed to examine the surface morphology of the MSNs. The dry MSN samples were placed onto double-sided carbon tape on an aluminium stub and then coated with gold (BAL-TEC SCD 050 sputter coater, Leica Microsystems, Wetzlar, Germany). 

The elemental composition of the MSNs was measured using Energy Dispersive X-Ray Spectroscopy (EDX; Bruker X-ray spectroscope with Edx Aztec software, Oxford Instruments, Abingdon, Oxfordshire, UK), across an image containing representatives of the different classes of the MSNs under SEM. These elemental compositions are subject to the assumption that the sample possessed a homogenous elemental composition and a flat surface in the range of the interaction volume of the primary electron beam. The parameters used during imaging included a scanning rate between 5 to 10-kilo counts per second, accelerating voltage of 20 kV and a working distance of between 5–10 mm. 

### 4.6. Nanoparticle Tracking Analysis (NTA)

MSN preparations in deionized water (100 μg/mL), were sonicated for ten minutes, and their hydrodynamic size and zeta potential analysed using NTA (NanoSight NS500 fitted with NTA software v3.0, Malvern Instruments Ltd., Worcestershire, UK). Prior to analysis, the system was flushed and primed, and the camera set at the zero position. The particle size distribution was based on their Brownian motion within the laser scatter volume and was calculated using the Stokes-Einstein equation. Zeta potential was calculated using the Smoluchowski approximation, based on laser-Doppler microelectrophoresis, with results presented as the mode ± standard error [40]. 

### 4.7. Nitrogen Adsorption and Desorption 

A Micrometrics Tri-Star II 3030 version 1.03 instrument (Micrometrics, Norcross, GA, USA), was used to obtain the nitrogen adsorption and desorption isotherms of the MSNs. Prior to analysis, the MSN was degassed at 363.15 K for 1 h, and at 473.15 K for 4 h, under nitrogen. The surface area was determined using the Brunauer-Emmet-Teller (BET) equation, and the pore volume by the single point method. The pore size distribution was obtained using the BJH model, together with the desorption branch of the isotherm [101]. 

### 4.8. Fourier Transform Infrared Spectrometer (FTIR)

Fourier transform infrared (FTIR) spectra were measured with a Bruker Alpha ATR Fourier Transform Infrared Spectroscopy (Bruker Billerica, MA, USA). All the samples were compressed into pellets and recorded at 64 scans from 4000 cm^−1^ to 400 cm^−1^ with a resolution of 4 cm^−1^.

### 4.9. Doxorubicin Release

Drug release was assessed using a standard protocol [40,73,102], over a 72-h period. Briefly, 5 mg of DOX-loaded MSNs were added to 3 mL of PBS at pH 4.2 and 7.4, respectively, with gently stirring at 37 ℃. Aliquots (100 μL) of the MSN suspensions were removed at intervals, centrifuged (13,000 rpm, 5 min) and the supernatant analyzed by UV-vis spectroscopy. Fresh PBS (100 μL) was added each time into the drug release solution to maintain sink volume. Experiments were conducted in triplicate and reported as means. The drug release percentage at each recorded time interval was analysed at 480/488 nm and calculated using the following equation [85]:

(9) $$\%\;R_{t}=\frac{C_{t}.V_{1}+V_{2}.\left(C_{t-1}+C_{t-2}+\ldots +C_{0}\right)}{W_{0}.L}\;\times \;100\;\%$$

where $C_{t}$ is the drug concentration at time interval t; $C_{t-1}+C_{t-2}$ are drug concentrations prior to time interval t ($C_{0}=0)$; $V_{1}$ is the total volume of the drug release bath (3 mL); $V_{2}$ is the volume extracted for UV-vis analysis (0.1 mL), $W_{1}$ is the initial weight of the DOX-loaded MSNs (0.005 g), and L is the drug loading capacity of the DOX-MSNs (taken from equation 8). 

### 4.10. MTT Cytotoxicity Assay

The cytotoxicity of the MSNs and their drug nanoconjugates in vitro was assessed using the 4,5- dimethylthiazol-2,5-diphenyltetrazolium bromide (MTT) assay [103] and methodology followed as per previous publications [40,78,87,104]. All cells were seeded in 96 well plates (containing 100 μL EMEM, 10%FBS and 10% antibiotics), at a density of 1 × 10^4^ cells/well and incubated at 37 ℃ in 5% CO_2_ for 24 h. Thereafter, the culture medium was replaced, DOX-MSNs (20, 50 and 100 μg/mL) added, and the cells incubated for 48 h. A positive control of untreated cells was included. The assay was conducted in triplicate. Following the incubation period, the medium was removed and replaced with 100 μL fresh medium containing 10% MTT (5 mg/mL in PBS) and incubated at 37 ℃ for 4 h. The MTT infused medium was then removed, and 100 μL of DMSO added to each well for solubilisation of the formazan crystals. The plates were gently shaken and absorbance measured at 540 nm using a Mindray MR-96A microplate reader (Vacutec, Hamburg, Germany). Cell viability (%) was calculated using the following formula:

(10) $$\%\;\mathrm{Cell}\;\mathrm{Survival}=\;\frac{A_{540\mathrm{nm}\;}\mathrm{of}\;\mathrm{treated}\;\mathrm{cells}}{A_{540}\;\mathrm{control}\;\mathrm{cells}\;\left(\mathrm{untreated}\right)}\;\times 100\;\%$$

### 4.11. Acridine orange/Ethidium bromide (AO/EB) Apoptosis Assay

The assay was conducted as previously described [105,106]. Cells were seeded into 24-well plates at a density of 1.5 × 10^5^ cells/well and incubated for 24 h. The medium was then replenished, and cells treated with the DOX-MSNs at pre-determined IC_50_ concentrations for 48 h, in triplicate, with untreated cells used as the control. Thereafter, the medium was removed, cells washed with PBS (2 × 200 μL), and stained with 12 μL/well of the dye solution (AO: EB, 1:1 *v*/*v* 1 mg/mL) for 5 min. Any excess dye was removed by washing cells with 200 μL of PBS. The stained cells were viewed using an Olympus inverted fluorescence microscope at 200× magnification, and images captured with a CC12 fluorescence camera (Olympus Co., Tokyo, Japan). Apoptotic indices were calculated according to the following equation:

(11) $$\mathrm{Apoptotic}\;\mathrm{Index}=\;\frac{\mathrm{Number}\;\mathrm{of}\;\mathrm{Apoptotic}\;\mathrm{Cells}}{\mathrm{Total}\;\mathrm{Number}\;\mathrm{of}\;\mathrm{Counted}\;\mathrm{Cells}}\;\times 100\;\%$$

### 4.12. Cell cycle Analysis 

Cells were seeded and treated with DOX-MSNs as in 4.11, according to a previously published protocol [40]. Following the 48 h incubation, cells were pelleted (300× *g* for 5 min), washed with PBS, resuspended in 200 μL ice-cold ethanol (70% *v*/*v*), and fixed by incubation at −20 ℃ overnight. The cells were then centrifuged and washed with PBS, followed by addition of 200 μL of Muse^®^ cell cycle reagent (propidium iodide, RNase A) to each sample and incubated for 30 min at room temperature in the dark. Samples were then analysed using a Muse^®^ Cell Analyzer and the associated Muse™ cell cycle software (Luminex Corporation, Austin, TX, USA). 

### 4.13. Statistical Analysis

All data were presented as mean ± SD (standard deviation). Statistical analyses were performed using ANOVA (one-way analysis of variance), (GraphPad Prism version 6, GraphPad Software Inc., San Diego, CA, USA). The Dunnett multiple comparison and Tukey honestly significant difference (HSD) tests were used as post hoc test comparatives between groups and a pre-set control, and across groups, respectively. P values less than 0.05 were regarded as significant. Dissolution kinetics parameters were evaluated using Microsoft Excel 2018™ (Microsoft, Redmont, WA, USA) and Excel Add-in DD Solver (Microsoft, Redmont, WA, USA) software.

## 5. Conclusions

The clinical appeal of nano-delivery strategies that can favourably encompass relatively potent and unstable hydrophobic drugs have become critical in nanomedicine. Hence, the demand for a multi-functional and easily manipulated nanomaterial such as MSNs is great. Furthermore, it has become cost-efficient to utilize materials that have already been fully characterized, and their pharmacokinetic fates assessed. In this study, a hydrophilic mesoporous matrix was designed for the optimal loading capacity of the hydrophobic drug, DOX. These MSNs showed optimal DOX loading and were able to release the drug with moderate efficacy, with no erosion or friction affecting the release behaviour. The drug formulation was highly biocompatible in normal cells and produced a substantial cytotoxic effect in the cancer cells tested. Overall, this formulation provided for reduced dosage concentrations, and dosing intervals necessary to elicit a therapeutic response, without producing detrimental effects in normal dividing cells. These results further alluded to the potential use of DOX-MSN nanoconjugate as a future therapeutic dosing regimen, with the MSN nano-vehicle capable of further synergistic applications, that can enhance the effectiveness of a single-free drug treatment. Overall, results obtained augur well for future in vivo applications of MSNs in drug delivery.

## Figures and Tables

**Table 1:** Size, PDI and zeta potential of all MSNs and DOX-loaded MSNs.

Nanoparticle	Mean Diameter (TEM) (nm ± SD)	PDI (SD/mean)^2^	Hydrodynamic Diameter (NTA) (nm ± SD)	PDI (SD/mean)^2^	Zeta Potential (mV)
MSN [40]	36.09 ± 7.08	0.0385	188 ± 51.6	0.0753	−9.8 ± 1
CMSN [40]	39.43 ± 7.22	0.0335	62.2 ± 16	0.0662	32.4 ± 0.4
2% PCMSN [40]	40.75 ± 7.11	0.0422	12 ± 3.3	0.0756	17.0 ± 16.5
5% PCMSN [40]	40.37 ± 7.70	0.0364	54.8 ± 2.1	0.0015	7.4 ± 0.7
2% PCMSN-DOX	59.98 ± 12.44	0.0430	93.0 ± 10.9	0.0137	0.4 ± 0.7
5% PCMSN-DOX	50.82 ± 10.40	0.0419	111.7 ± 38.2	0.1170	17.4 ± 0.1

**Table 2:** EDX Data obtained from scanning electron microscopy (SEM) images.

Nanoparticles	Wt%		Wt% Sigma	
MSN	Si	47.38	Si	0.51
	O	52.62	O	0.51
CMSN	Si	34.00	Si	1.26
	O	31.74	O	1.44
	C	34.26	C	2.25
2% PCMSN	Si	21.89	Si	0.17
	O	49.73	O	0.35
	C	28.38	C	0.44
5% PCMSN	Si	34.04	Si	0.27
	O	47.16	O	0.38
	C	18.80	C	0.56

**Table 3:** Loading Capacity of PCMSNs with DOX.

Doxorubicin Loaded MSNS		
	5% PCMSN	2% PCMSN
Loading capacity (%)	93.32	97.85
Loading capacity (MGDOX /MGMSN)	0.9332	0.9785

**Table 4:** IC_50_ values of DOX-MSN treatments administered to tested cell lines (from Figure 6).

Cell Line	2% PCMSN-DOX	5% PCMSN-DOX
HEK293	-	-
MCF-7	20 μg/mL	20 μg/mL
Caco-2	20 μg/mL	50 μg/mL
HeLa	50 μg/mL	100 μg/mL

## Supplementary Materials

The following are available online, Table S1: Correlation coefficients (R^2^) obtained from modelling DOX loaded 2% PCMSNs through release kinetic models at pH 7.4 and 4.2, Table S2: Correlation coefficients (R2) obtained from modelling DOX loaded 5 % PCMSNs through release kinetic models at pH 7.4 and 4.2, Table S3: Korsmeyer-Peppas model’s release exponent factor and corresponding Kopcha’s release model fitting results, Figure S1: Graphical representation of the kinetic release data.
