# Supplementary material for: Sterically Stabilised Polymeric Mesoporous Silica Nanoparticles Improve Doxorubicin Efficiency: Tailored Cancer Therapy
Source: Molecules. 2020 Feb 8;25(3):742. doi: 10.3390/molecules25030742 (PMC7037074; doi:10.3390/molecules25030742)
Supplement: Supplementary file 1 [file molecules-25-00742-s001.pdf]

Supplementary Information

Table S1: Correlation coefficients (R^2) obtained from modelling DOX loaded 2% PCMSNs through release kinetic models at pH 7.4 and 4.2.

pH	Zero-order	First - order	Higuchi's	Hixson-Crowell's	Korsmeyer-Peppas's	Kopcha's
**Correlation value (R^2)**						
4.2	0.52	0.79	0.90	0.86	0.86<br>n = 0.14	0.97
7.4	0.54	0.65	0.83	0.71	0.88<br>n = 0.20	0.95

n = release exponent (indicative of drug release mechanism).

Table S2: Correlation coefficients (R2) obtained from modelling DOX loaded 5 % PCMSNs through release kinetic models at pH 7.4 and 4.2.

pH	Zero-order	First - order	Higuchi's	Hixson-Crowell's	Korsmeyer-Peppas's	Kopcha's
**Correlation value (R^2)**						
4.2	0.45	0.55	0.91	0.80	0.95<br>n = 0.12	0.96
7.4	0.40	0.74	0.76	0.61	0.88<br>n = 0.16	0.97

n = release exponent (indicative of drug release mechanism).

Table S3: Korsmeyer-Peppas model's release exponent factor and corresponding Kopcha's release model fitting results.

pH 4.2				
**Multidrug formulation**	**Korsmeyer-Peppas Model**	**Kopcha Model**		
	n - value	A	B	A/B
2% PCMSN	0.14	14.55	2..12	6.86
5% PCMSN	0.12	12.06	1.79	6.74
**pH 7.4**				
**Multidrug formulation**	**Korsmeyer-Peppas Model**	**Kopcha Model**		
	n - value	A	B	A/B
2% PCMSN	0.20	11.70	1.63	7.18
5% PCMSN	0.16	21.94	3.00	7.31

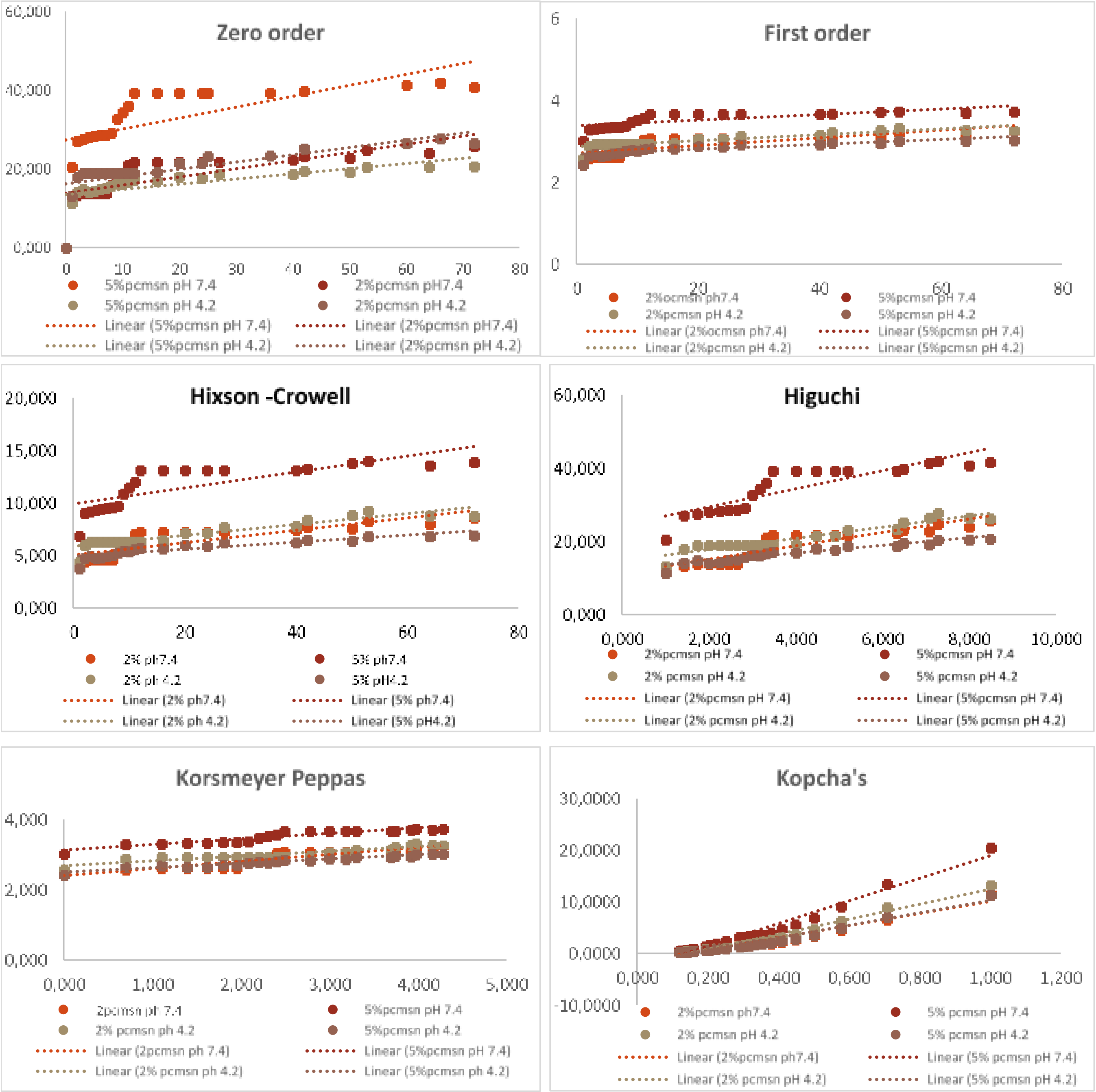

Figure S1: Graphical representation of the kinetic release data.
